# Nerve Lesions as the Origin of Certain Skin Diseases

**Published:** 1876-12

**Authors:** R. W. Westmoreland

**Affiliations:** Atlanta, Ga.


### NERVE LESIONS AS THE ORIGIN OF CERTAIN SKIN DISEASES.

By R. W. WESTMORELAND, M.D., Atlanta, Ga.

What I wish to suggest in this paper, and incline the members of the profession to discuss, is the feasibility of adopting an etiological classification of dermatological affections. In the present obscure and intricate arrangement of this class of diseases it will be apparent to the minds of all that a necessity exists for a system of nomenclature based upon *cause*, as distinguished from the present one founded on histological and physical changes.

Facts have been made prominent lately which lead those eminent in this branch of the profession to conclude that disturbance of certain nerves, which are denominated trophic, result in the phenomena of certain skin diseases. In this connection may be mentioned those affections known as eczema, erythema, psoriasis, lichen and pityriasis. These arise, it is affirmed, from lesions, either peripheral or centric, of those nerves supplying the parts concerned. It is manifest, for example, that in pruritus we have an abnormal condition of the nerves producing this troublesome symptom. And while we may have conditions of the blood directly leading to this disease, as in lithæmia, still the hypothesis of its nerve origin is demonstrated. Should, then, the condition of the blood be improved, the parts would return to their normal state, or the trouble, in a manner be relieved by remedies directed to the nerves.

Certain affections have been reported as more perfectly demonstrating this theory, such, for instance, as *herpes zoster*, consequent upon the reception of traumatic injuries and over exertion or other debilitating tendencies to the nervous system. Especially is this the case if a constitutional predisposition exists favoring the condition.

This theory is also supported in practice, for those diseases supposed to be from nervous origin often give way upon the administration of remedies directed to the nervous lesion. Prominent among these may be mentioned arsenic, strychnine and electricity. The efficacy of these has often been tested and approved by those experienced in the treatment of skin diseases.

At a recent discussion on this subject, Dr. Bulkley, an eminent dermatologist of New York, has this to say: "The relations of the nervous system to diseases of the skin were considered from a physiological and pathological point of view, with the following results:

1st. Microscopic anatomy shows the skin to be an organ remarkably well furnished with nerve filaments, their termini being associated with even the individual cells composing the *rete malpighii.*

2d. Physiology teaches that simple vaso-motor disturbance and nerve sections produce but slight and temporary changes. Attention is also directed to the reflex physiological relations of the skin, as flushing, blanching, etc., in deranged mental and bodily states.

3d. Pathology is found to be very rich in demonstrations of the direct connection between nerve influence and cutaneous phenomena, as shown in nerve lesion, and the pathological histology of certain skin diseases, notably *herpes zoster.*"

He then goes on to state his clinical experience, which further corroborates the neurotic relations of certain skin disturbances. In dermatological works the causes of many of the diseases treated of are found to be from impressions made upon some part of the nervous system. This is particularly the case in those affections called by Wilson erythemas, lichens, eczemas, etc., and which seem to be but a progressive intensity of the morbid condition, depending upon the greater or less nervous perturbation. Constitutional predispositions and reflex influence between parts in active sympathy are often the source or origin. As in uterine diseases we may have a chain of nervous symptons depending upon reflex or sympathetic action, so in gastric or intestinal disturbances we may have morbid changes taking place in the integument as a result of the active sympathy of these parts, and often exemplified in urticaria. The question then arises, upon what basis could we reasonably expect to build a classification referable to the neurotic orign of many of these troubles? While we may feel assured of their neurotic relations, the point to decide is the extent and character of the purturbation and the direct or indirect influence upon the integumentary nerves. To meet these distinctions we might make the first division into direct and indirect nervous influences. The first class would include such as depend upon immediate nervous impression, as from traumatic causes, or from conditions in which either general or local perturbation exists as the result of peripheral or centric disturbance. The second class would contain lesions, the result of diathesis, or constitutional affections in which the nervous system may be secondarily affected. As to the extent of the nerve lesion, producing corresponding intensity of perverted function, we may indicate the general or partial impressions as measuring or regulating the severity of the superficial disturbance.

We now arrive at the most important consideration of this subject, namely, the character and conditions of the physiological perturbation in the integument. From the fact, which is demonstrated by micropscopic anatomy and phosiological research, that the cutis and cuticle are abundantly supplied with nerve filaments, even to their most minute structure, it is reasonable to suppose that the state of their perfect or imperfect nutrition is controlled in accordance with the normal or abnormal condition of this nervous organism. Direct applications, for instance, such as cold or heat, under certain circumstances, interferes with the function of the part, while indirect impression gives rise to perturbations from sympathy with the other parts. The disturbance may primarily originate, too, from centric impressions on either the cerebro-spinal system or the ganglionic, as is affirmed by some authorities. In either event the integument suffers. Finally, as to the character of the perturbation giving rise to the integumentary manifestation. We find two conditions of nervous impressions, as those from irritation or debility, and such as arise from stimulation or exciting agents. The former are characterized by a congestive chronic type, while the latter assume, mainly, the inflammatory acute.

With this I submit the question. Should they induce the profession to the consideration of this subject, my chief desire will have been gratified. I did not expect to offer anything very important either in fact or theory, but started with the idea of exciting a discussion which would redound to the benefit of all. I might report cases in point, now under treatment, but as no very decided or satisfactory results have, up to this time, been obtained, they will be deferred. I will say, however, in conclusion, that I have been very much encouraged in treatment based upon the theory that I have attempted to present.

